# Microhabitat conditions drive uncertainty of risk and shape neophobic responses in Trinidadian guppies, *Poecilia reticulata*

**DOI:** 10.1002/ece3.10554

**Published:** 2023-09-25

**Authors:** Laurence E. A. Feyten, Indar W. Ramnarine, Grant E. Brown

**Affiliations:** ^1^ Department of Biology Concordia University Montreal Quebec Canada; ^2^ Department of Life Sciences The University of the West Indies St. Augustine Trinidad and Tobago

**Keywords:** antipredator behaviour, habitat complexity, habitat dimensions, information availability, neophobia, water velocity

## Abstract

In response to uncertain risks, prey may rely on neophobic phenotypes to reduce the costs associated with the lack of information regarding local conditions. Neophobia has been shown to be driven by information reliability, ambient risk and predator diversity, all of which shape uncertainty of risk. We similarly expect environmental conditions to shape uncertainty by interfering with information availability. In order to test how environmental variables might shape neophobic responses in Trinidadian guppies (*Poecilia reticulata*), we conducted an in situ field experiment of two high‐predation risk guppy populations designed to determine how the ‘average’ and ‘variance’ of several environmental factors might influence the neophobic response to novel predator models and/or novel foraging patches. Our results suggest neophobia is shaped by water velocity, microhabitat complexity, pool width and depth, as well as substrate diversity and heterogeneity. Moreover, we found differential effects of the ‘average’ and ‘variance’ environmental variables on food‐ and predator‐related neophobia. Our study highlights that assessment of neophobic drivers should consider predation risk, various microhabitat conditions and neophobia being tested. Neophobic phenotypes are expected to increase the probability of prey survival and reproductive success (i.e. fitness), and are therefore likely linked to population health and species survival. Understanding the drivers and consequences of uncertainty of risk is an increasingly pressing issue, as ecological uncertainty increases with the combined effects of climate change, anthropogenic disturbances and invasive species.

## Graphical abstract

In response to uncertain risks, prey may rely on neophobic phenotypes to reduce the costs associated with the lack of information regarding local conditions. We conducted an in situ field experiment of two high‐risk guppy populations designed to determine how the ‘average’ and ‘variance’ of several environmental factors might influence the neophobic response to novel predator models and/or novel foraging patches. Our results suggest neophobia is shaped by water velocity, microhabitat complexity, pool width and depth, as well as substrate diversity and heterogeneity, and we found that ‘average’ and ‘variance’ environmental categories differentially affected food‐ and predator‐related neophobia.

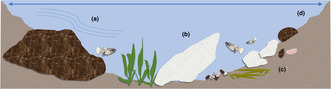

## 1 INTRODUCTION

Prey optimize their fitness by integrating and responding to publically available information (i.e. cues) in order to assess predation risk (Lima & Dill, 1990; McNamara & Dall, 2010). Some of these cues may be unreliable in that they do not consistently correlate with a particular event (Searcy & Nowicki, 2005). Examples of such unreliable cues include cues detected from a novel predator or a novel foraging opportunity, and prey lacking innate responses or prior experience with such unreliable novel cues would suffer from an inability to exhibit appropriate behavioural responses. Over time, prey may gain experience with these previously unknown cues, and learn this cue is associated with risk or a foraging opportunity. However, learning novel cues can be costly in terms of the time spent gathering information, and risking mortality when learning about a cue that conveys a predation risk (Dall et al., 2005; Ferrari et al., 2007; McNamara & Dall, 2010).

Neophobia, or the fear of novel stimuli, is thought to allow prey to respond to novel potential threats without the time and energy costs of learning, as well as the costs of potential mortality (Brown et al., 2013; Ferrari et al., 2015; Greenberg & Mettke‐Hofmann, 2001). However, individuals engaging in risk avoidance in response to novelty may miss possible novel foraging opportunities, as well as generally being unable to engage in other fitness‐enhancing activities like courtship or territory defence when engaging in risk averse behaviour (Lima & Bednekoff, 1999). Thus, a neophobic response should eventually be lost or retained as prey learn whether the novel cue indicates risk (Brown et al., 2015; Crane et al., 2020a; Feyten, Demers, Ramnarine, Chivers, et al., 2019). It has therefore been suggested that neophobia allows prey to deal with uncertainty of predation risk, or the inability to predict risk due to incomplete information (Brown et al., 2013; Feyten & Brown, 2018). Recent studies have demonstrated that neophobia is elicited under conditions of elevated predation risk (Brown et al., 2013; Feyten et al., 2022). Predator composition (diversity) also has an effect, albeit a weaker one (Feyten et al., 2022). Recent studies have also highlighted the importance of information availability and reliability in shaping neophobia (Feyten et al., 2021; Feyten, Demers, Ramnarine, & Brown, 2019; Feyten, Demers, Ramnarine, Chivers, et al., 2019).

**FIGURE 1:**
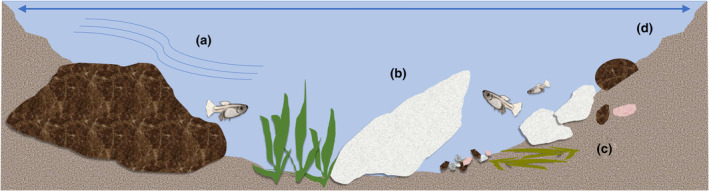
Variables within each microhabitat which may affect the availability of information to prey and predictability of risk, including (a) water velocity, (b) habitat complexity (i.e. rugosity), (c) substrate diversity and heterogeneity and (d) pool dimensions.

Information availability may be modified by habitat conditions (Weissburg et al., 2014), therefore we might expect neophobic responses in prey to be shaped by environmental conditions. This interference may be attributed to environmental ‘noise’, which has often been defined as background patterns or stimuli which interfere with the detection or response to cues of interest and which can occur in several modalities (Brumm, 2013; Koops, 1998; McNicol, 2004). However, noise may also be described as any unwanted or unintended additions to information, including distortions or transmission errors, which generate greater uncertainty (Shannon, 1948). Environmental conditions which generate noise may therefore interfere with risk assessment, leading to decreased predictability of predation risk and increased uncertainty of risk and neophobic responses (Figure 1). For example, increased stream velocity may contribute to uncertainty via its effect on the transmission (and detection) of chemical information in aquatic systems (Weissburg & Zimmer‐Faust, 1993). Similarly, the dimensions of a habitat have been suggested to affect the dilution of chemical information (Dickey & McCarthy, 2007), and regardless of modality, relevant information likely attenuates over distances (Weissburg et al., 2014) and needs to travel further to reach prey. As a result, prey in larger microhabitats may be left uncertain of risk if they are unable to invest sufficient time and energy into gathering information regarding risks. Moreover, larger and/or deeper local habitats may accommodate a larger density and diversity of predators (Harvey & Stewart, 1991; Tejerina‐Garro et al., 2005), resulting in less predictable (more uncertain) risk due to spatial and temporal variation in predation (Sih, 1992). For example, a recent study on guppies demonstrated that neophobic responses increased after experiencing both increased density and diversity of predation risk (Feyten et al., 2022).

Another likely environmental variable affecting information availability, and therefore uncertainty of risk, is microhabitat complexity (i.e. the structural or physical complexity within a habitat). Although microhabitat complexity may offer more refuge to prey and decrease encounter rates with predators (Gratwicke & Speight, 2005; Komyakova et al., 2013; Kovalenko et al., 2012; Nunes et al., 2015; Ross et al., 2007), it may also provide predators with refuge and impair visual or chemical information transmission (Dolinsek et al., 2007; Golub et al., 2005; Rilov et al., 2007). The latter is supported with evidence linking increased microhabitat complexity with increased predation success of ambush predators such as pike (*Esox lucius*; Greenberg et al., 1995) and bluegill sunfish (*Lepomis macrochirus*; Crowder & Cooper, 1982). A recent laboratory study on fathead minnows (*Pimephales promelas*) demonstrated that microhabitat complexity reduces baseline fear but failed to demonstrate a strong effect on neophobic responses (Crane, Ferrari, et al., 2020). However, this study used captive‐bred fish and did not assess the effect of complexity in a natural setting, and therefore may not accurately reflect natural levels of complexity experienced by this species or their behavioural responses in a more natural setting.

In addition to average values of these environmental variables, we also expect variability of these environmental factors (water velocity, microhabitat dimensions, and microhabitat complexity) within each microhabitat to decrease the predictability of risk (Koops, 1998). Behavioural flexibility has been suggested as beneficial in changing environments (Dunlap & Stephens, 2016; Reader, 2003; Sol, 2009; Tebbich & Teschke, 2014), and so we might expect changes to neophobic behavioural responses in response to variable environments. In the same vein, habitat substrate heterogeneity may shape the predictability of risk, with heterogeneous and variable habitats limiting information available to prey (Schmidt et al., 2010). This would likely impede risk assessment and result in less predictable risk, and therefore more uncertainty regarding risk, in heterogeneous habitats compared to homogeneous habitats. Alternatively, heterogeneous or diverse habitats may provide prey with more contextual clues in terms of physical landmarks, improving their spatial cognitive abilities when predicting where risk might occur (Schmidt et al., 2022; White & Brown, 2015) and resulting in reduced neophobia compared to prey from homogeneous habitats.

Previous studies have attempted to link habitat complexity and habitat variability to a variety of neophobic behaviours including activity in novel environments, neophobic foraging and object neophobia with mixed results (Aceves‐Fonseca et al., 2022; De Meester et al., 2021; Jenkins et al., 2021; Mettke‐Hofmann et al., 2002; Morand‐Ferron et al., 2019; Tebbich & Teschke, 2014). However, we are not aware of any studies linking the average and variance of specific habitat conditions with neophobia under contexts of elevated predation nor under natural conditions. In order to assess which environmental factors contribute to uncertainty and therefore neophobic responses in Trinidadian guppies (*Poecilia reticulata*), we conducted in situ field experiments to determine how substrate heterogeneity and diversity, and the average and variance of stream velocity, microhabitat dimensions and substrate complexity within a pool (see Section 3 of Methods for how these were defined and measured) might influence two measures of neophobic responses.

## 2 METHODS

### 2.1 Testing sites

We conducted in situ observations in a series of discrete pools, with a distance of at least 10 m between test pools, along an ~1‐km reach of the Lopinot River in April 2017 and the Acono River in April 2018 (Figure S1) in the Trinidadian Northern Range between 9:00 AM and 2:00 PM. A discrete pool is defined as a pool separated from others by fast‐moving ripples, waterfalls and/or rock boundaries, which limit the movement of guppies. Both the Lopinot and Acono rivers are known high‐predation sites, with abundant populations of pike cichlids (*Crenicichla* sp.), wolf fish (*Hoplias malabaricus*), blue acara (*Andinocara pulcher*) and brown coscarob (*Cichlasoma taenia*; Deacon et al., 2018). We conducted our experiments in both these sites in order to account for possible population‐specific effects. Both these populations experience elevated risk and therefore should exhibit neophobic behaviour (Brown et al., 2013). Indeed, neophobia in guppies originating from this site at the Lopinot has been well documented (Feyten et al., 2021; Feyten, Demers, Ramnarine, & Brown, 2019), and we expected this to be the case for the Acono population as well. We sampled 27 discrete pools (average length = 7.74 m, length range = 2.36–17.18 m) within the Lopinot River, and 15 discrete pools (average length = 7.14 m, length range = 3.93–14.34 m) in the Acono River.

### 2.2 Experimental protocol

Within each test pool, we assessed neophobic behaviours of guppies and environmental conditions, moving upstream in order to avoid testing a pool after disturbance had occurred upstream. First, we conducted two well‐established assessments of neophobic behaviour, in a randomized order, including latency to inspect a novel predator model, as well as latency to enter a novel foraging arena. Predator inspection behaviours are well documented assay of risk assessment (Brown et al., 2010; Dugatkin & Godin, 1992; Magurran & Seghers, 1990), with inspection behaviours allowing individuals to gather information regarding local predation threats. Inspection behaviours can be particularly risky when the predator is unknown (Fishman, 1999). Meanwhile, latencies to forage are widely documented and reviewed in the neophobic literature as risk‐averse behaviour (Crane, Brown, et al., 2020; Crane & Ferrari, 2017; Crane, Ferrari, et al., 2020; Greenberg & Mettke‐Hofmann, 2001; Mettke‐Hofmann et al., 2013). More specifically, our two behavioural assays were based on recent in situ experiments testing neophobia and perceived predation risk (Brown et al., 2013; Elvidge et al., 2016; Feyten et al., 2021; Feyten, Demers, Ramnarine, & Brown, 2019). We observed these behaviours three times each within each testing pool, such that each pool generally had six separate locations where behavioural assays were conducted. In order to avoid pseudo‐replication, we calculated an average of each response latency within each pool. We had three exceptions to this, where we were only able to sample latency to forage once in one pool, and twice in two pools. Guppies are usually confined to a pool during the dry season (Magurran, 2005), and it is therefore unlikely that individuals are moving to their preferred habitats. Thus, environmental conditions in each pool are likely shaping the observed guppy behavioural responses.

For latency to inspect a novel predator model, we placed our model in an area where at least five guppies were observed within a 50‐cm radius. The novel predator model was a 3D printed polycarbonate model sprayed with Rust‐Oleum® Painter's Touch® Ultra Cover Primer, hand‐painted with Pebeo® High Viscosity Studio Acrylic paint, and sealed with Rust‐Oleum® Painter's Touch® Ultra Cover Clear Gloss. The predator model was designed to represent a novel predator resembling a carp or goldfish painted in bright colours. A prior study using this model demonstrated that individuals from high‐risk sites respond with similar avoidance to a novel predator model compared to a predator model based on a known predator (Feyten, Demers, Ramnarine, & Brown, 2019). When integrating additional chemical information of varying reliability (i.e. known alarm cues and unknown novel cues) with the predator models, guppies respond differently depending on whether the model represents a known or novel predator, suggesting that individuals can distinguish between the models, but treat both as risky (Feyten, Demers, Ramnarine, & Brown, 2019). We attached the model to a wooden dowel (~1 m in length) using transparent fishing line, allowing controlled placement of the model such that it was placed on the substrate and fully submerged (Figure S2). We made observations from the shoreline, with observation sites in each pool located at least 1 m apart. As long as five guppies remained present within the 50‐cm radius, we started trials and recorded latencies of the first guppy or group of guppies to inspect the predator model, for a maximum trial time of 5 min. An inspection was defined as a guppy or group of guppies making a head‐first directed movement equivalent to one body length in distance towards the model (Brown et al., 2010; Dugatkin & Godin, 1992), when this movement was within five body lengths of the predator model. Risk‐averse behaviour is indicated by an increased latency to inspect (Brown et al., 2010, 2013).

For latency to enter a novel foraging arena, we gently placed a novel foraging arena (white corrugated plastic; ~30 × 25 × 23 cm) into stream pools at locations that visibly contained at least five guppies within a 50‐cm radius without visible currents or higher velocities. The arena was partially submerged (~10 cm depth), and a rock was placed inside to prevent the arena from moving due to the stream current. Prior to placement, a small amount (~0.5 g) of food (OMEGA™ One Freshwater Flakes, as in Feyten et al., 2021) was placed at the back of the foraging arena. The flakes were wetted before placing the arena gently into the pool in order to ensure the novel food stayed within, and at the bottom of, the arena. As long as five guppies remained present within a 50‐cm radius, we started trials and recorded the latency for guppies to enter the arena (crossing the dashed line; Figure S3), for a maximum trial time of 5 min. Risk‐averse behaviour was indicated by an increased latency to enter the novel arena (Elvidge et al., 2016; Feyten et al., 2021). We feel confident attributing increased latencies to enter a novel foraging arena to greater neophobic responses in higher complexity habitats, especially since the literature suggests complex environments (i.e. those with macrophytes) encourage exploratory behaviour in guppies under risk‐free laboratory conditions (Camacho‐Cervantes et al., 2015).

### 2.3 Environmental variables

Immediately after the behavioural assessments, we measured environmental conditions within the test pool. Environmental conditions measured included pool depth and width, pool length, water velocity, substrate complexity (rugosity) and substrate type. We measured pool depth five times: at the upper and lower parts of the pool, as well as the middle in the left bank, centre and right bank. We measured pool width thrice at the upper, middle and lower portion of the pool. We measured pool length once. We measured water velocity using a JDC Electronics Flowatch Flowmeter at the surface of the pool, as well as at 40% depth (mid‐depth) of the pool. These were measured five times, in similar fashion to pool depth. We measured substrate complexity (rugosity) by systematically placing a chain of 1 m up to five times in each pool, depending on pool size. We then measured the linear distance between the ends of the chain after it was placed on the substrate. We calculated the ratio of this linear distance to the original chain length of 1 m, and obtained a rugosity value by subtracting this ratio from 1 (Aronson & Precht, 1995; Leduc et al., 2007; see Figure S4). This resulted in a rugosity variable that increased with higher microhabitat complexity. We then calculated the average and variance of depth, width, surface velocity, mid‐depth velocity and rugosity for each pool (see Table S1 for complete list of environmental variables). There were six exceptions in the Lopinot River, where we failed to collect velocity data. We imputed the missing average and variance velocity values using the respective averages from the Lopinot population. We categorized the substrate type in a pool systematically up to five times, depending on pool size, using a grid frame (Figure S5). We categorized substrate type as sand, fine (<1 cm), coarse (1–3 cm), cobble (>3 cm), hard (smooth rock/granite) or leaf litter. We counted the number of grids (in 0.5 cm increments), out of the 25 total from the grid frame, corresponding to each substrate type. From these count values, we calculated several diversity measures. We calculated a measure of heterogeneity, Hurlbert's PIE (probability of interspecific encounter; Hurlbert, 1971) or the probability that two grid sections selected at random without replacement from a pool belong to different substrate categories. We also calculated alpha diversity (number of substrate types present) for each grid sample, and used the pool average of these alpha diversity values in the data analysis. We calculated our substrate metrics using the *hpie()* function from the benthos package (Walvoort, 2022) and the *specnumber()* function in the vegan package (Oksanen et al., 2022) using R‐Studio version 4.2.2. Larger values in substrate heterogeneity and average alpha diversity indicate potentially greater uncertainty in the environment. Given the nature of these metrics, we included these substrate measures in the ‘variance’ category in later analyses.

### 2.4 Statistical analyses

Given the large number of potential explanatory variables, we conducted data reduction using principal components analyses (PCAs) with the *PCA()* function from the FactoMineR package in R (Lê et al., 2008). We conducted PCAs on two environmental variable categories, ‘average’ and ‘variance’. Before conducting the PCAs, we checked the asymmetry and normality of our explanatory variables. We Box‐Cox transformed the explanatory variables which did not meet the assumption of normality in order to improve the symmetry of their distribution. We calculated the estimated transformation parameter (lambda) for each variable using *boxcoxfit()* from the geoR package in R (Ribiero et al., 2022). We added a small constant (0.0000001) to any variables which included zeros, namely the velocity variables. The environmental data were then standardized to account for the difference in unit measure (to get rid of the magnitude differences between variables), using *decostand()* in the vegan package of R (method = “standardize”; Oksanen et al., 2022). From each of the PCAs, we retained two principal components (PCs) to incorporate into further models. We decided to keep an equal number of components for each category in order to ensure average and variance of environmental variables had equal representation in our models, with a threshold of the components explaining at least 45% of the variance of our explanatory variables.

Once we extracted components from the average and variance PCAs, we included these components as explanatory variables in three global models. Our behavioural measures were not highly correlated (Kendall's *τ* = 0.17), so we conducted separate analyses for each. In order to control for potential population differences, we included population as a random effect in all our models. Given that the populations were not visited during the same year, this population term also served to control for potential effects associated with year. For latency to inspect a novel predator model, we conducted a global linear mixed model (LMM). For latency to enter a novel foraging arena, many trials had the maximal trial latency (i.e. guppies did not enter the arena within the 5‐min observation period). More specifically, no guppies entered the novel foraging arena in any of the replicated trials in roughly 21% of the tested pools. For this reason, we first conducted generalized linear mixed models (GLMMs) using a binomial distribution to determine how environmental variables might shape whether guppies entered the novel arena. We then followed with a LMM to determine how environmental variables might shape latencies to enter the novel arena for trials in which guppies did enter the novel arena. For both our LMMs, our models did not violate the assumption of normality of residuals or heteroscedastic residuals. We also checked multicollinearity of our explanatory components in each model using the *vif()* function from the car package (Fox & Weisberg, 2019). We used the lmer() or glmer() function from the lme4 package in R (Bates et al., 2015) our LMM and GLMM models, respectively.

We calculated criticality using bootstrapping to select the best model for our dataset, as well as determine which explanatory variables were the most critical to explaining the response (Azen et al., 2001). In order to assess criticality, we used the *dredge()* function from the MuMIn package in R (Bartoń, 2022), which selected the best models according to the corrected Akaike information criterion (AICc) for small sample sizes, with the difference (ΔAIC) > 2. In the case where multiple models had ΔAIC ≤ 2 in a replicate, the criticality calculation considered whether a variable was present in at least one of those models in that replicate. We bootstrapped using a sample size equivalent to the original experimental sample size (*n* = 42 for latency to inspect, *n* = 42 for binomial latency to enter and *n* = 33 for latency to enter when guppies did enter), with 1000 replications. All initial models included two explanatory components for average environmental variables, two explanatory components for variance environmental variables and population as a random effect.

All analyses were conduction using R‐Studio version 4.2.2.

## 3 RESULTS

### 3.1 Environmental principal components

Prior to conducting PCAs, we transformed several of our explanatory variables including pool length, width, width variance, depth, depth variance, rugosity, Hurlbert's PIE (heterogeneity), surface velocity and mid‐depth velocity. We used the transformed mid‐depth and surface velocity variables in our analyses, as their distributions were more symmetrical despite still differing significantly from the normal distribution (*p* < .01 for both). Similarly, the velocity variance metrics differed significantly from the normal distribution, but the data distribution was the most symmetrical without transformation, and therefore we used the untransformed variables in the subsequent PCA.

For the variation explained in environmental ‘average’ variables, we retained only the first two PCs for the reasons discussed above. Together, the first two PCs represented 58.23% of the total variance in the environmental data, the first representing 38.37% of the variation and the second 19.86% (Table 1a). The loadings, square loadings and contribution of each variable for the first two PCs are described in Table 2a. The variables contributing most to the first component were mid‐depth velocity, surface velocity, pool width, rugosity and depth (which had 75.60%, 64.34%, 29.53%, 28.78% and 23.25% of their variation explained by the first component, respectively; Table 2a), and this component is hereafter referred to as Velocity‐Complexity. Mid‐depth velocity, surface velocity and rugosity were all highly positively correlated with Velocity‐Complexity (0.8695, 0.8021 and 0.5365, respectively), while width and depth were negatively correlated with Velocity‐Complexity (−0.5434 and −0.4822, respectively; Figure S6). Thus, positive values in Velocity‐Complexity were characterized by high mid‐depth velocity, high surface velocity, and high rugosity, while negative values in Velocity‐Complexity were characterized by high pool depth and high pool width. The variables that contributed most to the second component were length and depth (which had 44.18% and 35.56% of their variation explained by the second component, respectively Table 2a), and this component is hereafter referred to as Pool Dimension. Length and depth were highly positively correlated with Pool Dimension (0.6647 and 0.5964; Figure S6). Rugosity and pool width were also positively correlated with Pool Dimension (0.3971 and 0.3078, respectively); however, these variables were poorly represented with each variable (having roughly 15% and 10% of their variation explained in this component, respectively). Positive values in Pool Dimension were thus characterized by high values of length and depth.

**TABLE 1:** Eigenvalues of the first three principal components given by PCAs for the (a) ‘average’ and (b) ‘variance’ environmental variable category.

	Principal Components		
	1	2	3
(a) PCA			
Eigenvalues	2.302	1.191	0.882
Cumulative Eigenvalues	2.302	3.494	4.3763
Cumulative proportion of total variation	38.37	58.23	72.94
(b) PCA			
Eigenvalues	1.917	1.410	1.055
Cumulative eigenvalues	1.917	3.327	4.382
Cumulative proportion of total variation	27.38	47.53	62.60

**TABLE 2:** Summary of the loadings (correlation with PC), square loadings (quality representation of variable within PC), and contribution of each response variable in the first two PCs for (a) ‘average’ and (b) ‘variance’ environmental variable categories.

Explanatory variables	Principal Components	
	1	2
(a) Rugosity (transformed)		
Loadings	**0.3536**	0.3637
Square loadings	0.2878	0.1577
Contribution	12.4998	13.2347
Pool length (transformed)		
Loadings	−0.1948	**0.6089**
Square loadings	0.0874	0.4418
Contribution	3.7966	37.0831
Pool width (transformed)		
Loadings	**−0.3581**	0.2820
Square loadings	0.2953	0.0948
Contribution	2.8254	7.9527
Pool depth (transformed)		
Loadings	**−0.3178**	**0.5463**
Square loadings	0.2325	0.3556
Contribution	10.0971	29.8480
Surface velocity (transformed)		
Loadings	**0.5286**	0.2743
Square loadings	0.6434	0.0896
Contribution	27.9460	7.5218
Mid‐depth velocity (transformed)		
Loadings	**0.5730**	0.2088
Square loadings	0.7560	0.0519
Contribution	32.8352	4.3597
(b) Rugosity variance		
Loadings	−0.1421	−0.1277
Square loadings	0.0386	0.0229
Contribution	2.0184	1.6246
Pool width variance (transformed)		
Loadings	**−0.3622**	**0.5356**
Square loadings	0.2515	0.4047
Contribution	13.1192	28.6899
Pool depth variance (transformed)		
Loadings	−0.1729	**0.6069**
Square loadings	0.0573	0.5195
Contribution	2.9890	36.8324
Substrate alpha diversity		
Loadings	**0.5598**	−0.1678
Square loadings	0.6006	0.0397
Contribution	31.3359	2.8146
Substrate Hurlbert's PIE (transformed)		
Loadings	**0.5572**	0.3295
Square loadings	0.5950	0.1531
Contribution	31.0420	10.8576
Mid‐depth velocity variance		
Loadings	**0.4030**	0.3681
Square loadings	0.3113	0.1911
Contribution	16.2431	13.5504
Surface velocity variance		
Loadings	0.1803	0.2373
Square loadings	0.0623	0.0794
Contribution	3.2523	5.6305

*Note*: In bold are the variable loadings of interest (where the square loadings ≥0.23) for the components retained for subsequent models.

For the variation explained in environmental ‘variance’ variables, we retained only the first two PCs. Together, the first two PCs represented 47.53% of the total variance in the environmental data, the first representing 27.38% of the variation and the second 20.15% (Table 1b). The loadings, square loadings and contribution of each variable for the first two PCs were described in Table 2b. The variables contributing most to the first component were average alpha diversity and substrate heterogeneity, as well as mid‐depth velocity variance and pool width variance (which had 60.06%, 59.50%, 31.13%, and 25.15% of their variation explained by the first component, respectively; Table 2b). This component is hereafter referred to as Substrate Diversity. Average alpha diversity, substrate heterogeneity and mid‐depth velocity variance were highly positively correlated with Substrate Diversity (0.7750, 0.7714 and 0.5580, respectively), while pool width variance was negatively correlated with Substrate Diversity (−0.5015; Figure S7). Thus, positive values in Substrate Diversity were characterized by high average alpha diversities, high substrate heterogeneity and high mid‐depth velocity variation, while negative values in Substrate Diversity were characterized by high pool width variance. The variables that contributed most to the second component were pool depth variance and pool width variance (which had 51.95% and 40.47% of their variation explained by the second component, respectively; Table 2b). This component is hereafter referred to as Dimension Variance. Pool depth variance and pool width variance were highly positively correlated with Dimension Variance (0.7207 and 0.6361, respectively; Figure S7). Mid‐depth velocity variance and substrate heterogeneity also contributed to this component, and were correlated positively with it (0.4372 and 0.3913, respectively), but were poorly represented with 19.11% and 15.31% of their variation explained (Table 2b). Positive values in velocity variance were thus characterized by high values of pool depth variance and pool width variance.

### 3.2 Latency to inspect

**FIGURE 2:**
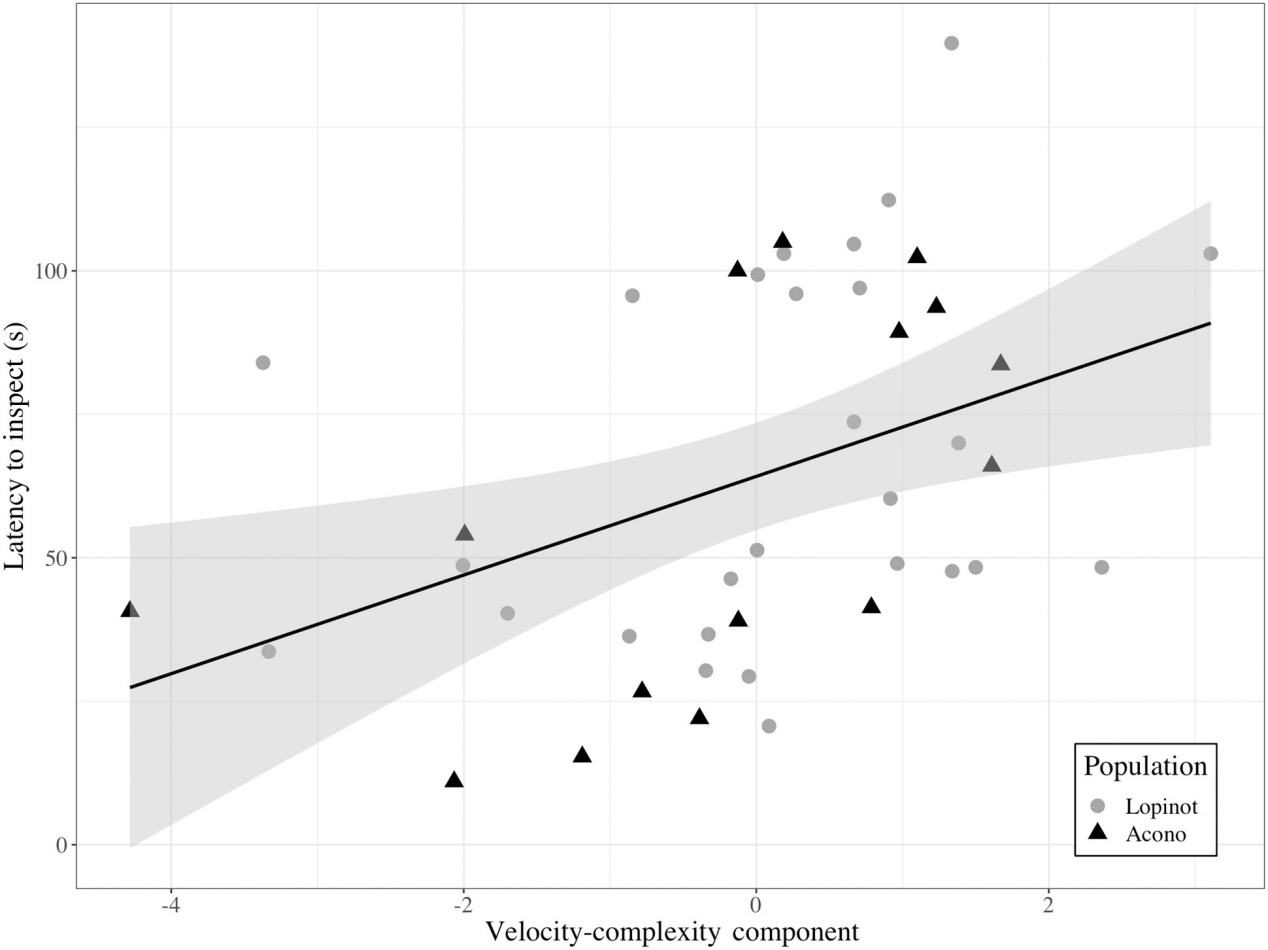
Relationship (±SE) between the latency to inspect a novel predator model (s) and the Velocity‐Complexity component. The shape and colour of the data points indicate whether the observation came from the Lopinot (grey dot) or the Acono (black triangle) population.

Our GLMM revealed that the Velocity‐Complexity component best explained the latency to inspect a novel predator model, where an increase in Velocity‐Complexity resulted in increased latencies to inspect (*p* = .0158, df = 37, *t* = 2.530; Figure 2; Table S2). In other words, guppies increased their latencies to inspect a novel predator model (i.e. were more neophobic) when their microhabitats had high mid‐depth and surface velocities, complex substrate, as well as shallow and narrow dimensions. Criticality bootstrapping revealed that all four components were highly critical (i.e. each component was present in the best model, or at least one of the best models when ΔAIC ≤ 2 in a replicate, in 100% of replicates). It also revealed that all four components were represented across the best fitting models for each replicate, in all replicates (Table S4a).

### 3.3 Latency to enter

We found that none of our components significantly explained whether guppies entered a novel foraging arena (*p* > .21 for all components; Table S3). Our criticality assessment indicated that only the Velocity‐Complexity component was highly critical (i.e. it was present in the best model, or at least one of the best models when ΔAIC ≤ 2 in a replicate, in >75% of replicates). It also revealed that all four components were represented across the best fitting models within each replicate, in the highest proportion of the replicates (19.1%; Table S4b) compared to other variable combinations.

**FIGURE 3:**
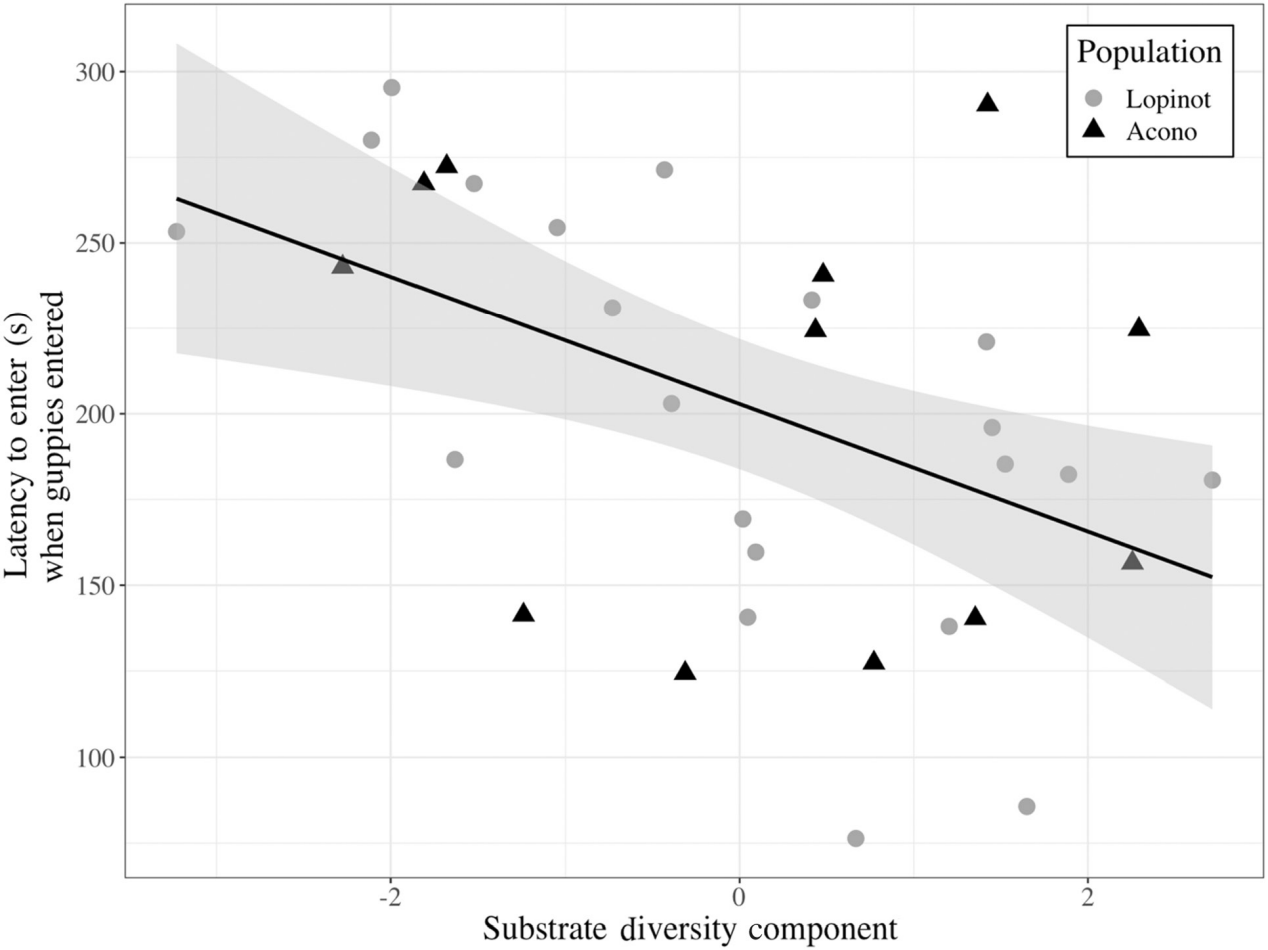
Relationship (±SE) between the latency to enter, when guppies entered, a novel foraging arena (s) and the Substrate‐Diversity component. The shape and colour of the data points indicate whether the observation came from the Lopinot (grey dot) or the Acono (black triangle) population.

The LMM on latencies to enter a novel foraging arena, in trials where guppies did enter, revealed that our Substrate Diversity component best explained when guppies entered, where an increase in Substrate Diversity resulted in decreased latencies to enter (*p* = .0013, df = 28, *t* = −3.574; Figure 3; Table S5). In other words, guppies were quicker to enter a novel foraging arena in microhabitats with larger values in substrate heterogeneity and average substrate alpha diversity. Criticality bootstrapping revealed that all included components were highly critical (i.e. each component was present in the best model or at least one of the best models when ΔAIC ≤ 2 in a replicate, in 100% of replicates). It also revealed that all four components were represented across the best fitting models within each replicate, in all replicates (Table S4c).

## 4 DISCUSSION

Our results suggest that several environmental variables, including water velocity, microhabitat complexity, pool width and depth, as well as substrate diversity and heterogeneity, shape neophobic behaviour (i.e. latencies to inspect a novel predator model, and latencies to enter a novel foraging arena) in guppies from high‐risk environments. Therefore, our results suggest that these environmental variables influence risk perception and likely shape uncertainty of risk experienced by prey. Interestingly, we found that the ‘average’ and ‘variance’ categories differentially affected our two neophobic metrics. One of the ‘average’ category components shaped neophobic predator inspection behaviours, whereas one of the ‘variance’ category components shaped how prey engage with novel foraging opportunities. This highlights the importance of examining the effects of both ‘average’ and ‘variance’ environmental categories, as demonstrated in a recent work by Sánchez‐Tójar et al. (2020), which is generally lacking in the existing literature. Moreover, our results highlight that environmental variables can affect neophobia differently depending on the context (e.g. predator‐related neophobia and food‐related neophobia). Future studies of neophobia should continue to incorporate multiple contexts of neophobic responses. Although we found no effect of population in any of our analyses, we kept them in our models as a control for genetic differences and observation year. Given the lack of population effect, we could not confirm any differences between the two study populations, suggesting that our results can be generalized to other guppy populations experiencing similar risk conditions.

For latency to inspect a predator model, we found a significant positive effect of the Velocity‐Complexity component. In other words, an increase in water velocity and microhabitat complexity, as well as a decrease in pool width and depth, resulted in greater latencies to inspect a novel predator model. The results for water velocity and microhabitat complexity fall in line with our predictions, where habitats with higher water velocity may result in chemosensory information that is often unavailable or quickly diluted (Weissburg & Zimmer‐Faust, 1993). Since guppies use chemosensory alarm cues to assess risks in their environments, higher velocities likely result in individuals unable to reliably assess risk in their environments. As a result, individuals should generally be more uncertain of risk, and thus exhibit greater neophobia when faced with novel cues. Additionally, increased velocity potentially increases turbidity, which may interfere with the perception of visual risk information. Although we did not measure turbidity levels in our study, turbidity was uniformly low and did not differ visibly across our testing pools. Thus, visual risk information was unlikely to be affected by water velocity in our study, but this relationship should be noted for future studies. Similarly, in habitats with elevated substrate complexity (i.e. habitat complexity), prey may be more uncertain of risk given visual obstruction (i.e. noise) in their environment. As a result, when microhabitats contain noise in the form of water velocity or substrate complexity, prey may generally be less likely to predict risk if their ability to perceive cues at any given time is decreased. Thus, in such environments under conditions of elevated risk, prey likely experience elevated uncertainty of risk and exhibit greater neophobia in order to err on the side of caution. Furthermore, given that individuals can use sensory complementarity to integrate cues from multiple modalities to more accurately assess risk and reduce uncertainty (Munoz & Blumstein, 2012), microhabitats which interfere with at least one sensory modality should restrict such complementation and likely increase the uncertainty of risk experienced by prey, and therefore their neophobic responses.

Unlike our predictions that increased habitat dimensions may lead to greater uncertainty, we found that decreased habitat dimensions (width and depth) resulted in greater latencies to inspect novel predator models. Although deeper and larger pools generally have a greater number and diversity of predators (Harvey & Stewart, 1991; Tejerina‐Garro et al., 2005), perhaps shallower and narrower pools may decrease prey ability to escape, especially given that guppy movement between discrete pools is seasonally limited. Moreover, shallow pools may increase exposure to other guppy predators such as the semi‐aquatic fishing spiders (including *Dolomedes* sp. and *Ancylometes bogotensis*), which situate themselves at the edge of a pool and use water surface disturbances and physical contact with dorsal fins to detect and capture prey (Bleckmann & Lotz, 1987; Deacon et al., 2015; Nyffeler & Pusey, 2014). Similarly, guppies in shallower pools may be more exposed to aerial predators such as fishing bats (*Noctilio leporinus*), which can rake pool surfaces with their claws or detect ripples at the waters' surface using echolocation (Brooke, 1994; Magurran, 2005). An increased risk of mortality in such smaller confined habitats may have caused individuals to err on the side of caution by expressing greater neophobia in response to a novel predator model. Another possible explanation is that larger habitats hold greater densities of conspecifics, which may provide a single individual with a greater probability of survival when all are under the threat of predation (i.e. safety in numbers; Lehtonen & Jaatinen, 2016). Although we ensured a minimum number of guppies were present before starting a trial, we did not measure guppy densities within each pool. Furthermore, increased guppy density may provide individuals with additional social information regarding risk or safety levels. Indeed, guppies use social information to inform their risk assessment, shaping their response to novelty (Brown et al., 2013; Crane, 2017). For example, guppies in high‐risk environments also increasingly use disturbance cues (i.e. a cue released after disturbance from a predator or disruption) from familiar conspecifics to inform their antipredator decisions (Crane et al., 2020b). Moreover, guppies can perceive ‘safety’ from calm conspecifics (Crane, 2017) and have been shown to integrate these safety cues to reduce their neophobia towards a novel foraging arena (Feyten et al., 2021). Therefore, we cannot discount that the observed decrease in neophobic predator inspection behaviours in larger pools may have been shaped by guppies (a) being more informed (i.e. less uncertain) about general risk conditions due to increased availability of ‘safety’ or ‘risk’ social cues or (b) having greater perceived safety due to safety in numbers or availability of social safety cues.

For latency to enter a novel foraging arena, there were no good predictors for whether guppies entered. This result may be limited by the fact that since guppies failed to enter in only 21% of replicate trials, there may not have had enough variation in environmental data in the ‘failed to enter’ group. However, there was a significant negative effect of the Substrate Diversity component on latencies in trials where guppies did enter. Thus, somewhat surprisingly, increases in substrate heterogeneity and average substrate alpha diversity resulted in decreased neophobia. Perhaps, from a long‐term standpoint, more heterogeneous environments promote ecosystem stability (Brown, 2003; Schmidt et al., 2022) and consequently create more predictable risk. Schmidt et al. (2022) argue that when habitats have greater heterogeneity, individuals are more able to discriminate between options. In contexts of predation, perhaps greater substrate heterogeneity and diversity promote prey ability to accurately assess location‐specific risk. Potentially, prey in such habitats may be generally more certain of risk, spatially, and less likely to exhibit neophobia. Indeed, guppies exhibit lower neophobia when risk is spatially predictable compared to spatially unpredictable (Feyten, Demers, Ramnarine, Chivers, et al., 2019). This is in line with results from another study which demonstrated that multiple species of gobies from more homogeneous habitats were slower to learn about reward locations and did not use visual landmarks as much as gobies from stable, more heterogeneous, and complex habitats (White & Brown, 2015). Furthermore, our results provide evidence of how habitat heterogeneity and complexity, which are often used interchangeably in the literature (Kovalenko et al., 2012), may exert differing effects on behaviours of interest. In our study, although substrate complexity resulted in an increase of one measure of neophobia, substrate heterogeneity resulted in a decrease of another neophobic measure.

It is important to note that the effects of habitat complexity may not be straightforward. For example, habitat complexity has been shown to decrease anxious behaviour in zebrafish (*Danio rerio*; DePasquale et al., 2016), fearful behaviour in fathead minnows (Crane, Ferrari, et al., 2020), and flight initiation distances in reef fishes (Chan et al., 2019; Nunes et al., 2015). In another study on the turquoise killifish (*Nothobranchius furzeri*), fish reared in complex environments were demonstrated to be less risk‐averse in novel habitats, but more risk‐averse in open habitats (Thoré et al., 2020). Thus, barren environments may result in elevated baseline fear if individuals have no refuge. There is likely an amount of habitat complexity that is the most advantageous based on the size of both prey and predator in that it provides refuge for small prey, without providing refuge for larger predators or obstructing information transfer (Almany, 2004; Crane, Ferrari, et al., 2020), potentially leading to greater uncertainty. If there is some optimal amount of habitat complexity for prey, the relationship between complexity and neophobia may not be a positive linear one. Although we did not assess a non‐linear relationship between habitat complexity and neophobia in our study, it may be worth exploring how the scale of prey and predator compared to refuge size and habitat complexity size shapes this relationship in several study systems. Additionally, open habitats lead to more efficient foraging of pursuit predators and complex habitats lead to greater success of ambush predators (Beukers & Jones, 1998; Crowder & Cooper, 1982; Greenberg et al., 1995), thus predator foraging tactics may also influence how habitat complexity shapes neophobia. For example, guppies have been demonstrated to exhibit different antipredator responses based on the predator species experienced (Botham et al., 2006, 2008). Future studies should therefore account for predator foraging tactics when assessing the effects of microhabitat complexity on prey behaviour.

Although we did not explore the cognitive mechanisms for our demonstrated link between habitat variables and neophobia, the literature suggests that environmental variables such as habitat complexity and heterogeneity shape cognitive spatial abilities. Several taxa of fish have been shown to have lower spatial learning and memory when raised in barren unenriched environments compared to when raised in complex enriched environments (Bergendahl et al., 2016; Carbia & Brown, 2019; Makino et al., 2015; Roy & Bhat, 2016; Salvanes et al., 2013; Zhang et al., 2021). Even variation in enrichment location and novel object presentation enhanced learning (DePasquale et al., 2016). As discussed above, gobies from stable spatially complex habitats were quicker to learn about reward locations and used both visual landmarks and turn directions, whereas gobies from dynamic homogeneous low‐complexity habitats made more errors and relied more on turn direction to orient themselves (White & Brown, 2015). This enhanced cognition and behavioural flexibility may be reflected with larger brain sizes (Kotrschal et al., 2013). Enhanced cognition and behavioural flexibility associated with larger brains may allow individuals to better cope with changing environments (i.e. uncertainty; De Meester et al., 2021; Sol, 2009). If individuals from complex environments have increased cognitive abilities and are more behaviourally flexible, then neophobia may be an additionally adaptive behavioural response between initial encounter with novelty and learning. Prey from high‐risk and high‐complexity environments may exhibit increased neophobia upon initial detection of novel information, and paired with increased cognitive abilities should be better able to learn and remember spatial risk compared to individuals in simple environments. Greater cognitive ability and behavioural flexibility may allow prey to quickly learn whether a novel cue is risky, and neophobia can rapidly be extinguished or can lead to rapid learning of the spatial nature of threat (Brown et al., 2015; Crane, Brown, et al., 2020; Feyten, Demers, Ramnarine, Chivers, et al., 2019). Thus, fish from high‐complexity environments may be better equipped to deal with novel risks, such as invasive predators, compared to their low‐complexity counterparts.

Future studies should also assess how other environmental conditions may contribute to uncertainty of risk and neophobia by interfering with information transmission. In aquatic environments, both turbidity and acidification would interfere with prey reception of visual and chemical cues, respectively, and may result in increased neophobic responses. For example, pH affects freshwater snail (*Physa acuta*) responses to olfactory predator cues (Cothran et al., 2021), and increased acidification increases antipredator responses to visual predator cues in Atlantic salmon (Elvidge et al., 2013). As such, we may expect acidification to play a role in uncertainty of risk, shaping neophobic responses in the absence of complementary visual information. Moreover, turbidity and acidity may both be present and interact to shape antipredator responses and neophobia. We suggest that future studies should carefully consider which neophobic metrics are used to assess these effects, given that turbidity has been demonstrated to decrease social behaviours such as shoaling, and a decrease in activity may be an effective antipredator strategy in turbid conditions (Borner et al., 2015). Future studies should also assess how environmental conditions might shape the availability of auditory information, such as vegetation and ambient noise (Weissburg et al., 2014) or anthropogenic noise pollution (Corcoran & Moss, 2017). These future studies should consider that environmental variables which generate noise in one modality may also interfere with antipredator responses in other modalities (Morris‐Drake et al., 2016). Additionally, neophobia may increase due to the variability and unpredictability of risk in an environment stemming from seasonality, habitat degradation, and urbanization (Ferreira & Faria, 2021; Jenkins et al., 2021; Morand‐Ferron et al., 2019).

To our knowledge, our study is the first to directly assess the link between neophobia and habitat complexity, in addition to other environmental variables, in situ. Our results have demonstrated the importance of studying both the average and variance of environmental variables on neophobic responses in two high‐predation populations of the Trinidadian guppy. Our research adds to the theoretical framework of how uncertainty of risk shapes neophobia and arises due to prey experience with information and predator composition (Crane, Brown, et al., 2020; Feyten et al., 2022; Feyten, Demers, Ramnarine, & Brown, 2019; Feyten, Demers, Ramnarine, Chivers, et al., 2019). Our results are consistent with our hypothesis that habitat variables may interfere with information transfer (i.e. environmental noise) and therefore increase uncertainty of risk, shaping the strength of neophobic responses in prey. Future studies should attempt to directly quantify information availability (e.g. chemical information) under different microhabitat conditions in order to directly link information availability, which shapes uncertainty of risk experienced by prey, to neophobia. It is important to understand in which contexts prey may be neophobic, as it is an adaptive response to the potential introduction of novel, invasive predators.

## AUTHOR CONTRIBUTIONS

**Laurence E. A. Feyten:** Conceptualization (equal); data curation (equal); formal analysis (lead); funding acquisition (equal); methodology (equal); writing – original draft (lead); writing – review and editing (equal). **Indar W. Ramnarine:** Project administration (equal); resources (equal); writing – review and editing (equal). **Grant E. Brown:** Conceptualization (equal); data curation (equal); funding acquisition (equal); investigation (equal); methodology (equal); project administration (equal); resources (equal); supervision (lead); writing – review and editing (equal).

## FUNDING INFORMATION

This work was supported by funding to L.E.A.F. by the Fonds de recherche du Québec (FRQNT) via a doctoral scholarship, as well as to G.E.B. by Discovery Grants from the Natural Sciences and Engineering Research Council of Canada.

## CONFLICT OF INTEREST STATEMENT

The authors declare no conflicts of interests.

## PERMISSION TO REPRODUCE MATERIALS FROM OTHER SOURCES

None.

## Supporting information


Appendix S1.

Data S1.

## Data Availability

Analyses reported in this article can be reproduced using data and code from Dryad Digital Repository: https://doi.org/10.5061/dryad.jm63xsjhd.
