# Supplementary material for: Microhabitat conditions drive uncertainty of risk and shape neophobic responses in Trinidadian guppies, Poecilia reticulata
Source: Ecol Evol. 2023 Sep 25;13(9):e10554. doi: 10.1002/ece3.10554 (PMC10518753; doi:10.1002/ece3.10554)
Supplement: Supplementary file 1 — Appendix S1. [file ECE3-13-e10554-s001.pdf]

Supplemental Materials

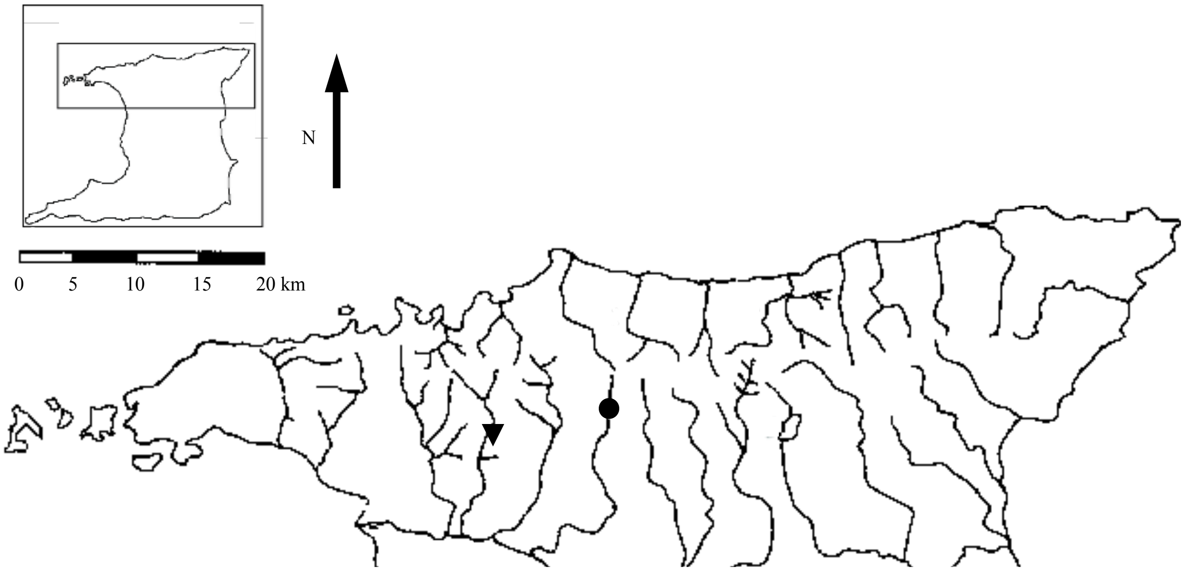

Figure S1. Map showing the location of our study site on the Acono (triangle) and Lopinot (dot) rivers in the Northern Range, Republic of Trinidad and Tobago.

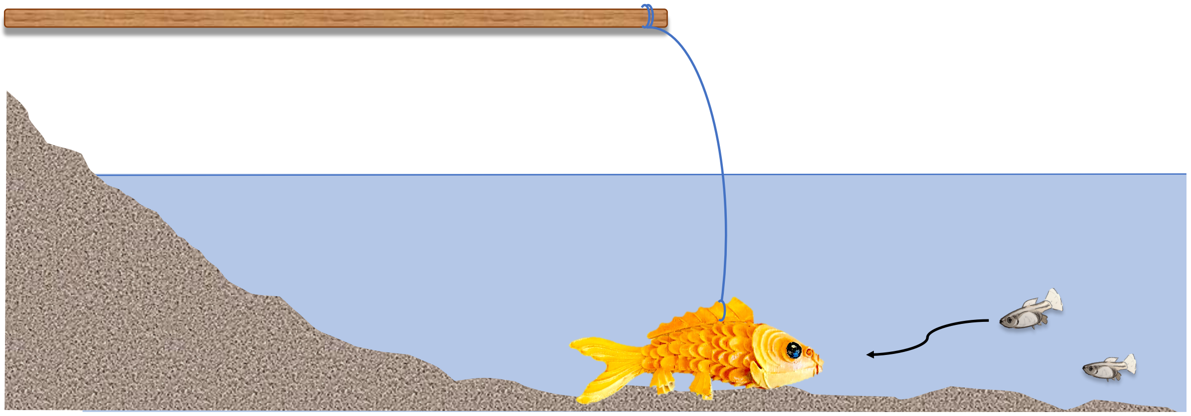

Figure S2. Schematic diagram of the 3D printed novel heterospecific predator model, 14 cm in length, attached to a dowel and placed gently along the banks of each testing pool.

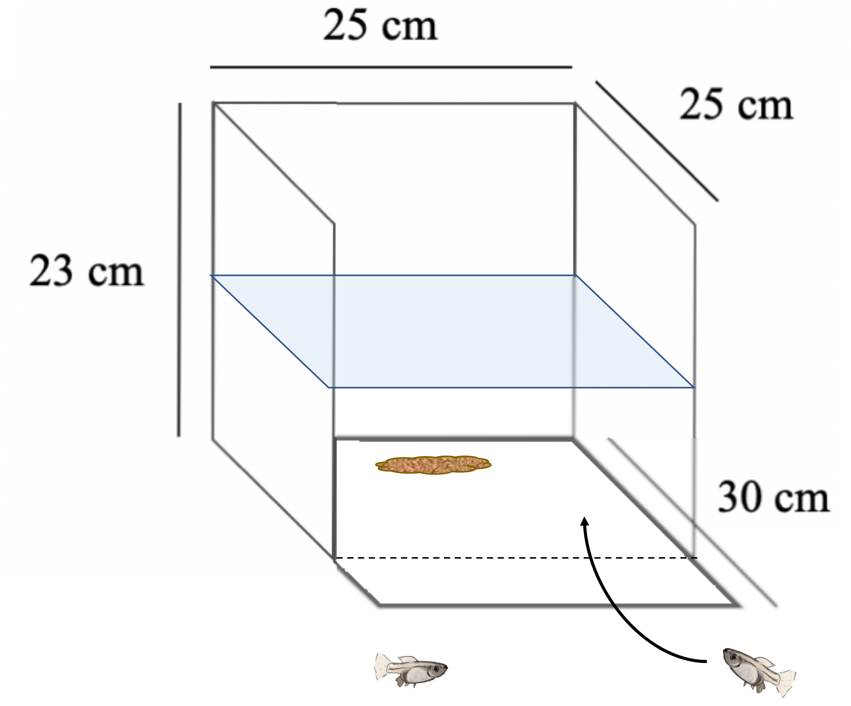

Figure S3. Schematic of novel foraging arena used for measuring latencies to enter a novel foraging arena, showing the delineation determined as an entry into the arena (dashed line) and the novel food (fish flakes) inside.

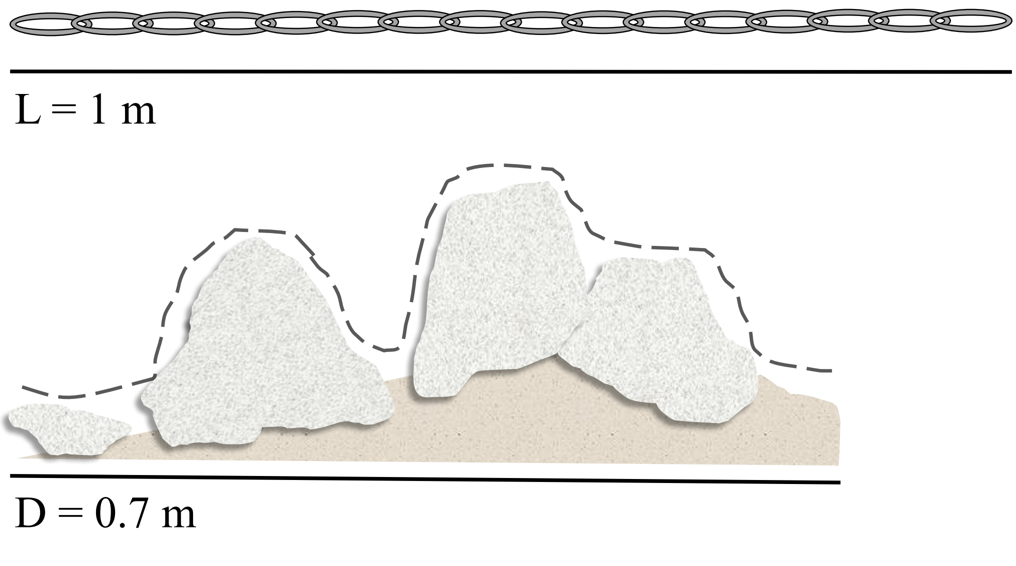

Figure S4. Schematic of rugosity measure, where a chain of original length (L) of 1 m was placed in a pool, and the linear length of the chain was measured (D). Rugosity was calculated as 1 - D/L. In this example, Rugosity would equal 0.3.

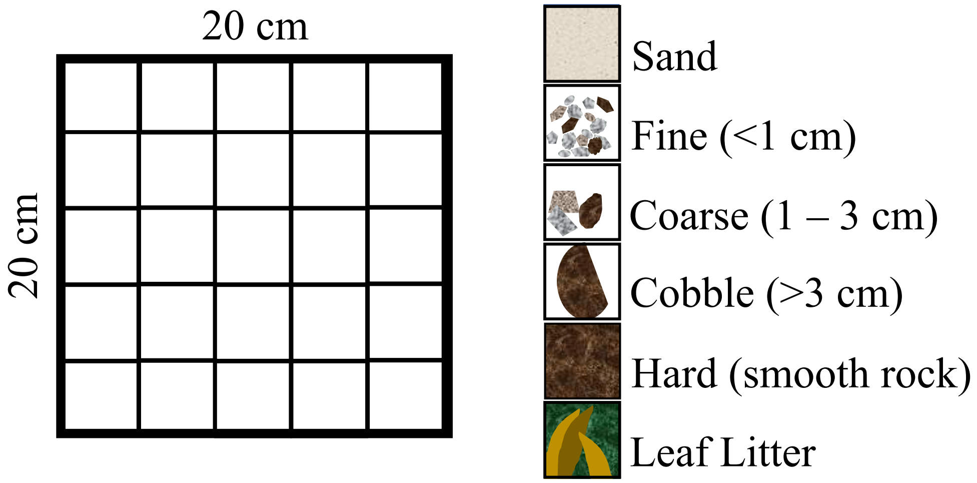

Figure S5. Schematic of the grid frame (20 cm × 20 cm) used for testing, along with the substrate classifications used.

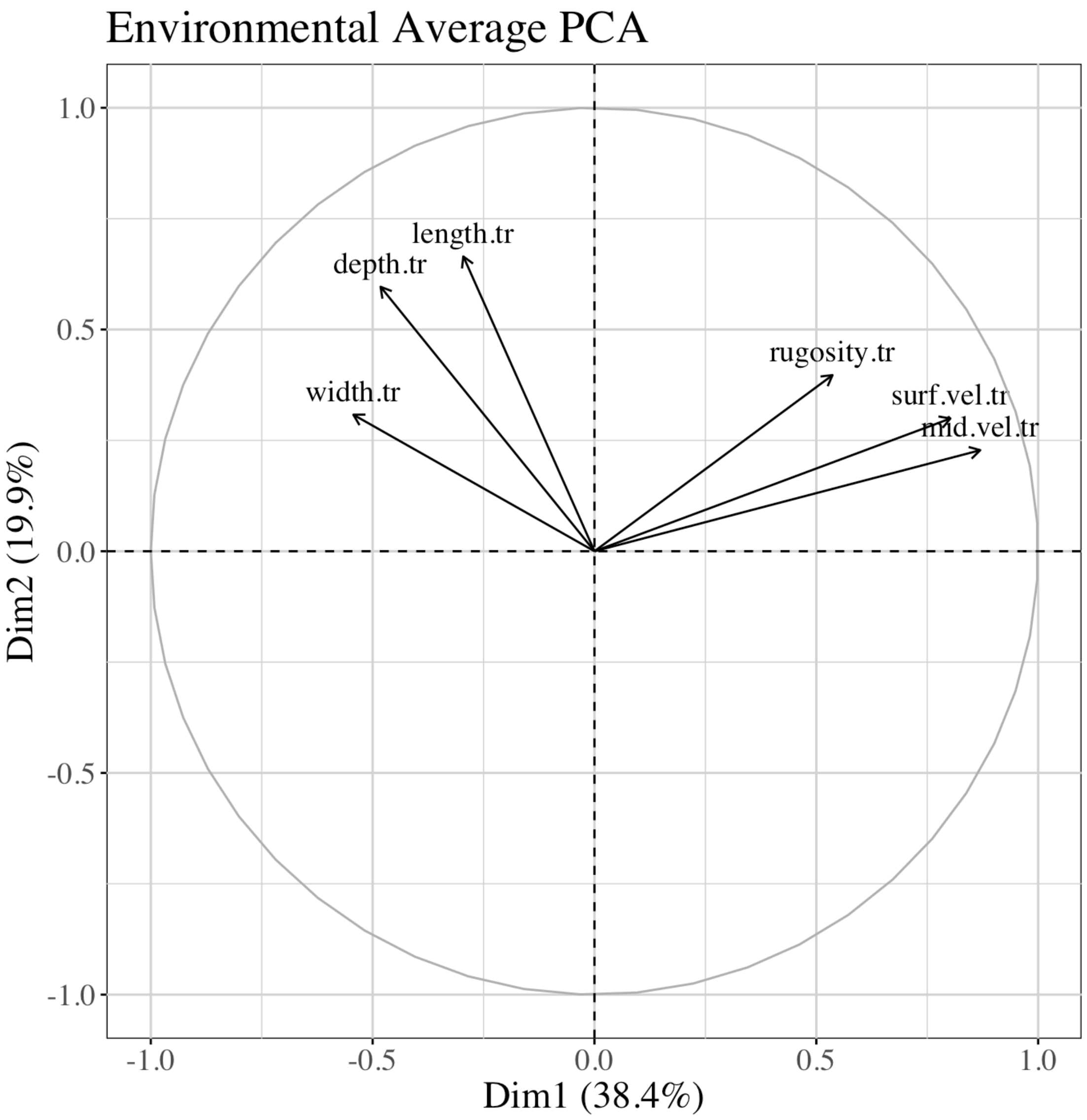

Figure S6: Biplot in Scaling Type 2 of the "average" environmental variables in PC1-PC2.Variables include the transformed average pool width (width.tr), transformed average pool depth (depth.tr), transformed pool length (length.tr), transformed average substrate complexity (rugosity.tr), transformed average surface water velocity (surf.vel.tr), and transformed average mid-depth water velocity (mid.vel.tr).

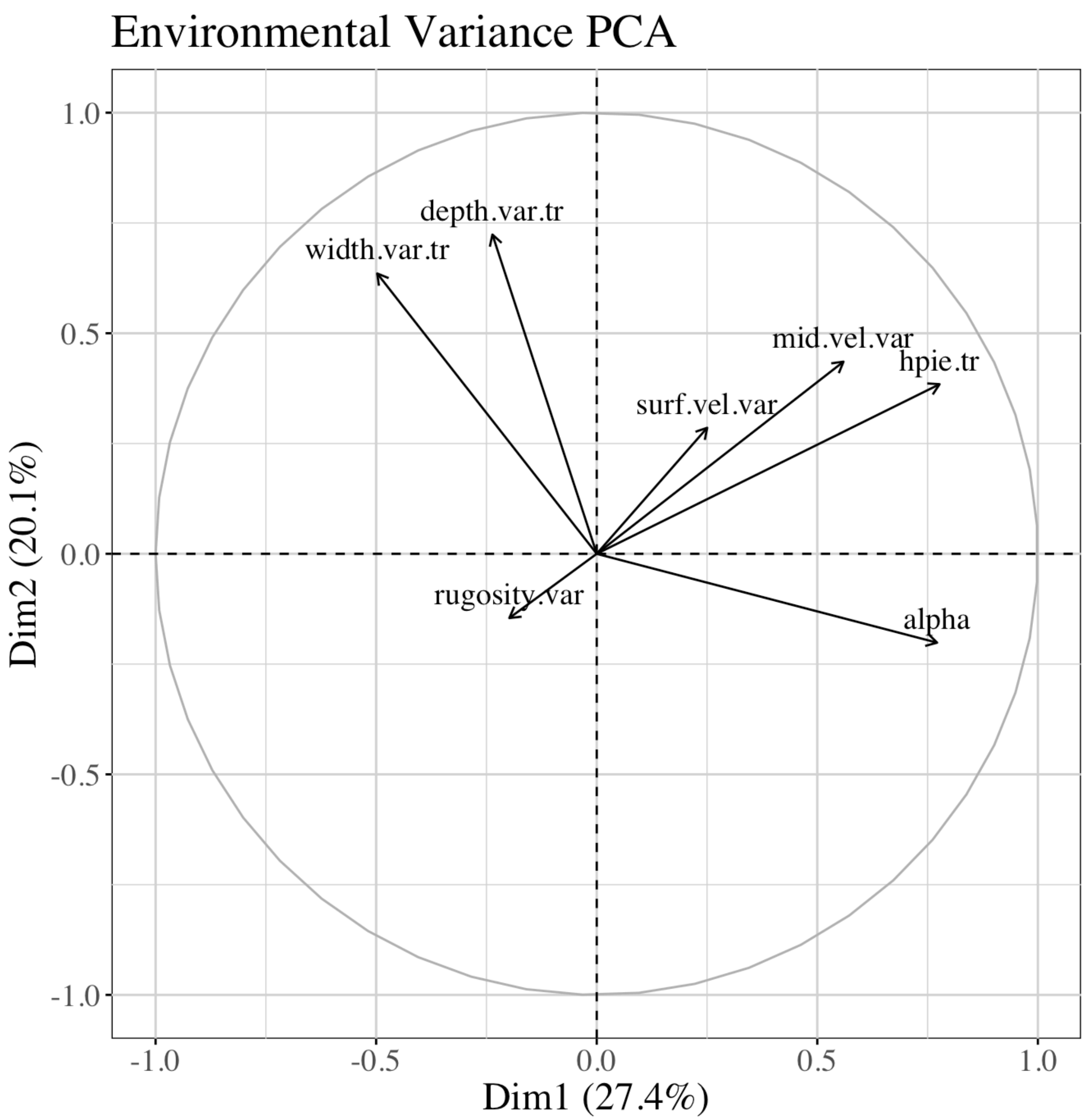

Figure S7: Biplot in Scaling Type 2 of the “variance” environmental variables in PC1-PC2.Variables include transformed pool width variance (width.var.tr), transformed pool depth variance (depth.var.tr), substrate complexity variance (rugosity.var), surface water velocity variance (surf.vel.var), and mid-depth water velocity variance (mid.vel.var), transformed substrate heterogeneity (hpie.tr), and substrate alpha diversity (alpha).

Table S2: Linear mixed-effect model output for the latency to inspect a novel predator model, including Satterthwaite t-tests on (a) fixed effects, and (b) statistics for random effects. Velocity-Complexity and Pool Dimension correspond to the first and second components of the "average" environmental variable category, respectively. Substrate Diversity and Dimension Variance correspond to the first and second components of the "variance" environmental variable category, respectively. Significant terms are in bold type. Degrees of freedom and p values estimated using Satterthwaite's approximations.

Condition	*Estimate*	*SE*	*df*	*t*	*p*
a) Fixed Effects					
Intercept	64.183	4.566	37	14.056	**<0.0001**
Velocity-Complexity	7.769	3.070	37	2.530	**0.0158**
Pool Dimension	7.527	4.789	37	1.572	0.1245
Substrate Diversity	5.394	3.502	37	1.540	0.1320
Dimension Variance	-0.8938	4.255	37	-0.210	0.8348
b) Random Effects				*Variance*	*SD*
Population (Intercept)				0	0
Residual				875.7	29.59
Marginal R^2 / Conditional R^2	0.2286 / 0.2286				

Model equation:

Latency to Inspect ~ Velocity-Complexity + Pool Dimension + Substrate Diversity + Dimension Variance + (1 | Population)

Model includes 42 observations and 2 populations, fit using REML

Table S3: Generalized linear mixed-effect model (Binomial distribution) output for whether guppies entered a novel foraging arena, fit by maximum likelihood (Laplace approximation) on (a) fixed effects, and (b) statistics for random effects. Velocity-Complexity and Pool Dimension correspond to the first and second components of the "average" environmental variable category, respectively. Substrate Diversity and Dimension Variance correspond to the first and second components of the "variance" environmental variable category, respectively. Significant terms are in bold type.

Condition	*Estimate*	*SE*	*z*	*p*
a) Fixed Effects				
Intercept	1.4317	0.4224	3.390	**0.0007**
Velocity-Complexity	-0.3844	0.3121	-1.232	0.2181
Pool Dimension	-0.2526	0.3982	-0.634	0.5258
Substrate Diversity	0.1321	0.2936	0.450	0.6528
Dimension Variance	0.3748	0.3684	1.017	0.3090
b) Random Effects			*Variance*	*SD*
Population (Intercept)			0	0

Theoretical Marginal R^2 / Conditional R^2 0.1330 / 0.1330
Delta Marginal R^2 / Conditional R^2 0.0783/ 0.0783

Model equation:

Binary Enter ~ Velocity-Complexity + Pool Dimension + Substrate Diversity + Dimension Variance + (1 | Population), family= binomial(link = "logit"))

Model includes 42 observations and 2 populations, fit using REML

Table S4: Criticality of explanatory variables (PCA components) across all replicates using bootstrapping with 1000 iterations for a) Latency to Inspect, b) Latency to Enter (Binomial), and c) Latency to Enter (in trials where guppies entered). 'Proportion' indicates the proportion of replicates which contained a particular combination of components across their respective best model(s) (according to ΔAIC).

	Explanatory Components					
	Velocity-Complexity	Pool Dimension	Substrate Diversity	Dimension Variance	n	Proportion
a)	1	1	1	1	1000	1.000
Criticality	1.000	1.000	1.000	1.000		
b)	1	1	1	1	191	0.191
	1	1	0	1	166	0.166
	1	0	1	1	112	0.112
	1	0	0	1	95	0.095
	1	1	1	0	71	0.071
	1	1	0	0	71	0.071
	1	0	0	0	59	0.059
	1	0	1	0	58	0.058
	0	1	1	1	47	0.047
	0	1	0	1	35	0.035
	0	0	1	1	24	0.024
	0	0	0	1	21	0.021
	0	1	1	0	15	0.015
	0	1	0	0	12	0.012
	0	0	0	0	12	0.012
	0	0	1	0	11	0.011
Criticality	0.823	0.608	0.529	0.691		
c)	1	1	1	1	1000	1.000
Criticality	1.000	1.000	1.000	1.000		

Table S5: Linear mixed-effect model output for the latency to enter a novel foraging arena, in trials where guppies entered, including Satterthwaite t-tests on (a) fixed effects, and (b) statistics for random effects. Velocity-Complexity and Pool Dimension correspond to the first and second components of the "average" environmental variable category, respectively. Substrate Diversity and Velocity Variance correspond to the first and second components of the "variance" environmental variable category, respectively. Significant terms are in bold type. Degrees of freedom and p values estimated using Satterthwaite's approximations.

Condition	*Estimate*	*SE*	*df*	*t*	*p*
a) Fixed Effects					
Intercept	202.888	9.060	28	22.395	**<0.0001**
Velocity-Complexity	8.622	5.657	28	1.524	0.1387
Pool Dimension	-12.493	9.856	28	-1.269	0.2149
Substrate Diversity	-22.552	6.311	28	-3.574	**0.0013**
Dimension Variance	14.240	9.207	28	1.547	0.1332
b) Random Effects				*Variance*	*SD*
Population (Intercept)				0	0
Residual				2661	51.58
Marginal R^2 / Conditional R^2	0.3237/ 0.3237				

Model equation:

Latency to Enter ~ Velocity-Complexity + Pool Dimension + Substrate Diversity + Dimension Variance + (1 | Population)

Model includes 33 observations and 2 populations, fit using REML
